# Upcycling Secondary Cellulosic Fibres and Post-Industrial Recycled Polypropylene from Household Appliance Manufacturing into Circular Composites with Enhanced Dimensional Stability

**DOI:** 10.3390/polym18212659

**Published:** 2026-10-27

**Authors:** Carmen Martínez-García, Vesna Žepič Bogataj, Ana B. López, Simon Kotnik, Francisco J. Iglesias-Godino, Manuel Valverde-Ibáñez, María Teresa Cotes-Palomino

**Affiliations:** 1 Department of Chemical, Environmental and Materials Engineering, Higher Polytechnic School of Linares, University of Jaén, Campus Científico-Tecnológico de Linares, 23700 Linares, Jaén, Spain; ablopez@ujaen.es (A.B.L.); figodino@ujaen.es (F.J.I.-G.); mvalver@ujaen.es (M.V.-I.); mtcotes@ujaen.es (M.T.C.-P.); 2 Slovenian Tool and Die Development Centre (TECOS), Kidričeva Cesta 25, SI-3000 Celje, Slovenia; vesna.zepic.bogataj@tecos.si; 3 Gorenje Gospodinjski Aparati, Partizanska Cesta 12, SI-3320 Velenje, Slovenia; simon.kotnik@gorenje.com

**Keywords:** recycled polypropylene, cellulosic fibres, composites, thermal properties, dimensional stability

## Abstract

This study addresses the development, thermal characterisation, and dimensional stability of circular composites based on a post-industrial recycled polypropylene (rPP) matrix from household appliances. The matrix was reinforced with natural (B600 and BC300) and recycled (SIS250, PWC500, and ZZC500) cellulosic fibres at 10%, 20%, and 30% dosages, using 3% MAPP as a compatibilizer. Processing was conducted via twin-screw extrusion and injection moulding. Systematic evaluations (TGA-DTG, DSC, and DIL) showed a minor reduction (2.6–5.1%) in thermal degradation onset, maintaining a safe processing window. Cellulosic fibres generally promoted matrix crystallization through heterogeneous nucleation, increasing matrix crystallinity up to 39.3% in composite B600-30 compared to 20.4% for the reference rPP matrix. Furthermore, the reinforcements acted as dimensional stabilizers, reducing the linear thermal expansion coefficient (α_40−120_) by up to 28.9% (ZZC500-30) while increasing thermal conductivity by up to 32.7% (B600 series). This approach demonstrates a viable upcycling strategy, yielding high-secondary-content circular composites by combining post-industrial recycled polypropylene matrices with reinforcements (virgin wood cellulose benchmarks (B600 and BC300), pre-consumer recycled cellulose (PWC500), post-consumer recycled cellulose (ZZC500), and recycled technical sisal (SIS250), complemented by 3 wt.% commercial MAPP compatibilizer.

## 1. Introduction

In line with the European Strategy for Plastics in a Circular Economy and Spanish Law 7/2022 on waste, transforming post-industrial household appliance polypropylene and secondary cellulosic waste into technical-grade composites provides a scalable pathway toward decarbonisation and material circularity. Among polyolefins, polypropylene (PP) has become one of the most widely used polymers due to its versatility, low cost and excellent mechanical properties, finding extensive applications in sectors ranging from packaging to the automotive industry. However, this widespread use has also created a major waste-management challenge, as the volume of generated plastic waste continues to exceed current recycling capacities. In this context, the transition towards a Circular Economy requires plastic waste to be transformed from an environmental burden into a valuable “second-life” raw material, thereby reducing dependence on virgin resources and minimising the industrial carbon footprint [1,2].

This transition is driven not only by ethical and environmental considerations but also by increasingly stringent regulatory frameworks at both European and national levels. In Spain, Law 7/2022 on waste and contaminated soil has introduced taxation measures targeting non-reusable plastic packaging, thereby compelling industries to significantly increase the incorporation of recycled polymers into their products. The achieved 28.9% reduction in linear thermal expansion (α_40−120_) directly addresses the strict dimensional tolerances required by the EU Sustainable Products Initiative and Eco-design Directives for durable household appliance components. This evolving legislative framework has a particularly strong impact on strategic sectors such as packaging, where material recyclability has already become a key market requirement, and the household appliance industry, which, in line with the European Union’s eco-design directives, is actively seeking to replace virgin materials with high-performance green composites [3,4]. In this context, the valorisation of polypropylene and cellulosic fibre waste into new secondary raw materials not only contributes to reductions in environmental impact but also supports the economic and regulatory sustainability of essential industrial products [5].

Although mechanical recycling remains the most widely implemented recycling strategy, the industry continues to face the inherent degradation of material properties associated with repeated processing cycles. Post-industrial polypropylene frequently exhibits variations in melt flow index, accompanied by reductions in molecular weight and structural integrity, limiting its reuse in high-value technical applications. To address these limitations, current research has increasingly focused on the development of fully recycled composites, in which not only is the polymer matrix recovered but reinforcing phases derived from secondary waste streams, such as cellulosic fibres, are also incorporated. This approach to total material circularity seeks to effectively close the technical lifecycle of materials by transforming multiple waste streams into a new generation of high-performance composite materials [6,7].

The valorisation of these materials through the development of ‘green composites’ enables sustainability objectives to be achieved without compromising technical performance. By incorporating recycled reinforcements into recovered polypropylene matrices, it is possible not only to restore but also to enhance the properties of the original virgin polymer, thereby providing a viable pathway towards the decarbonisation of the polymer processing industry [8,9].

Replacing virgin polymers with recycled materials in high-tech industrial applications presents significant engineering challenges that extend beyond conventional waste-management practices. For a material to be considered “high-performance” within the framework of a circular economy, it must exhibit dimensional stability and mechanical properties comparable to or even exceeding those of its fossil-based counterparts. However, recycled polypropylene (rPP) commonly undergoes thermo-oxidative degradation as a consequence of previous life cycles and shear stresses generated during reprocessing. This degradation results in polymer chain scission, leading to reduced impact resistance and diminished thermal stability [10]. One of the most widely used strategies to mitigate these limitations involves the development of new composites incorporating recovered reinforcing materials aimed at restoring the structural integrity and functional properties of the degraded polymer matrix [11,12,13,14].

Furthermore, the industrial viability of these fully recycled composites depends largely on their performance under real service conditions [15,16,17]. As highlighted by Bute et al. [18], understanding key parameters such as thermal conductivity and the coefficient of linear thermal expansion is essential for predicting the long-term durability and dimensional stability of components. In this context, the incorporation of recovered functional fillers not only contributes to reductions in production costs but also enhances and tailors material properties, enabling composites derived entirely from waste streams to comply with the stringent performance and safety requirements imposed on mass-market consumer products [2,19].

The investigation of thermal properties is fundamental for predicting both the processability and the in-service performance of composite materials developed entirely from recovered sources. In systems where both the polypropylene (rPP) matrix and the reinforcing phase are recycled, thermogravimetric analysis (TGA) represents a key initial step in assessing thermo-oxidative stability. This technique enables the definition of a safe processing window by identifying the onset degradation temperatures of the recovered reinforcing materials, which generally exhibit lower thermal stability than their virgin counterparts [10,20]. In addition, differential scanning calorimetry (DSC) plays a crucial role in the analysis of phase-transition phenomena, allowing the determination of crystallinity, melting temperature and crystallisation behaviour—parameters that strongly influence mechanical stiffness and cooling kinetics during industrial injection moulding processes [2].

Beyond their behaviour during industrial processing, the technical viability of these composites for engineering applications also depends on their response to temperature fluctuations under service conditions [15]. In this context, the coefficient of linear thermal expansion (α) is a key indicator of dimensional stability, as optimised α values help prevent deformation, warpage, and structural failure in technical components exposed to thermal cycling [18]. Thermal conductivity (λ) further complements the thermal characterisation profile and is particularly important when these green composites are intended for applications in sectors such as construction, household appliances, and electronics. According to the studies carried out by Saffian et al. [21] and Bhuiyan et al. [7], the incorporation of recycled fibres enables the modulation of heat-transfer behaviour, enhancing the materials’ thermal insulation capability and thereby supporting the use of fully recovered resources in high-performance engineering applications.

Progress towards true sustainability in materials engineering relies on the development of composites in which both the polymer matrix and the reinforcing phase are derived from waste streams, thereby achieving full technical circularity. In this context, the use of recycled polypropylene (rPP) as the matrix, combined with recycled reinforcements such as the pre-consumer and post-consumer cellulosic fibres investigated in this study, represents one of the most effective strategies for reducing the carbon footprint associated with technical materials.

As reported by Bhardwaj et al. [9], the incorporation of recovered cellulose fibres into polymer matrices not only enhances the tensile and storage moduli but can also provide mechanical performance comparable to that of composites based on virgin polymers, provided that adequate fibre dispersion and a critical fibre length are maintained throughout the recycling and processing stages.

The principal challenge associated with fully recycled composites arises from the cumulative degradation of both the polymer matrix and the reinforcing phase. Recycled polypropylene (rPP) commonly undergoes reductions in molecular weight and melt viscosity as a consequence of repeated thermal and mechanical stresses experienced during previous processing and service cycles. Similarly, recycled cellulosic fibres may exhibit diminished reinforcing efficiency due to fibre shortening, structural damage, and morphological alterations induced by repeated mechanical processing [10]. Previous studies by Poletto [16] and Xie et al. [17] have demonstrated that that interfacial compatibility is a key factor governing the overall performance of such composite systems. In this regard, the incorporation of functional coupling agents is essential in promoting interfacial adhesion between the non-polar rPP matrix and the polar cellulosic reinforcement. The formation of chemical and physical interactions at the interface enhances stress transfer efficiency, improves thermal stability and contributes to the overall integrity of the composite. Consequently, optimisation of the fibre–matrix interface enables recycled composites to obtain mechanical and thermal properties suitable for demanding engineering applications while maintaining the processability required for industrial manufacturing processes such as injection moulding [8,19].

Furthermore, the comprehensive thermal characterisation of these recycled binary systems enables the prediction of their behaviour under real service conditions. Thermo-oxidative stability, evaluated through thermogravimetric analysis (TGA), and crystallisation kinetics, analysed by differential scanning calorimetry (DSC), are significantly influenced by the presence of recycled fillers, which may act as a nucleating agents, thereby modifying the degree of crystallinity and, consequently, the stiffness and thermal performance of the final material [20,22]. By employing exclusively recovered components, it is possible not only to reduce the environmental impact associated with conventional plastics but also to valorise waste streams that would otherwise be disposed of in landfills. This demonstrates that the combination of recycled polymer matrices and recovered reinforcements represents a technically viable approach for engineering applications requiring a balanced combination of sustainability, dimensional stability and thermal functionality [7,18].

In light of the challenges outlined above, the primary objective of this research is to develop and comprehensively characterise a series of green composites based on a recycled polypropylene (rPP) matrix reinforced with cellulose-based fibres of different origins, including virgin fibres, as well as pre-consumer and post-consumer recycled fibres. To this end, multiple material formulations were developed in order to evaluate the influence of both fibre origin and reinforcement content on the thermal behaviour by means of TGA-DSC, as well as on dimensional stability and heat-transfer performance, assessed through the coefficient of linear thermal expansion and thermal conductivity, respectively.

As highlighted by Saffian et al. [21], a thorough understanding of these thermal and dimensional properties is essential to ensure that recycled material can perform reliably and safely under real service conditions. Ultimately, this research aims to demonstrate that high-performance composite materials can be successfully developed from recovered resources through the combined use of recycled polymer matrices and virgin, pre-consumer and post-consumer cellulose-based fibres. In this way, the study contributes to the decarbonisation of the manufacturing industry by promoting the replacement of virgin fossil-based materials with circular and recyclable alternatives aligned with the sustainability and efficiency objectives of the modern bioeconomy [1,8].

## 2. Materials and Methods

### 2.1. Materials

The raw materials used for the manufacture of the composites consisted of a polymer matrix, reinforcing fibres and a coupling agent.

The polymer matrix was ground recycled polypropylene (rPP) obtained from a single stream of post-industrial waste generated by GORENJE IPC (Velenje, Slovenia) during the manufacture of household appliances. The material originated from a controlled and homogeneous production process, and all experiments were performed using material from the same production batch to minimise batch-to-batch variability. It should be noted that the post-industrial rPP matrix was a heterogeneous polymer compound containing approximately 30 wt.% inorganic mineral fillers (predominantly talc and calcium carbonate, as confirmed by TGA residue analysis). In addition, minor residual traces of cylindrical glass fibres were detected in the matrix stream, originating from prior processing runs of glass-fibre-reinforced appliance components at the recycling facility [23].

The reinforcing phase comprised five distinct cellulose-based fibre grades supplied by J. Rettenmaier & Söhne GmbH & Co. KG (JRS, Rosenberg, Germany), encompassing both virgin and secondary raw materials to investigate the effect of fibre origin and morphology on composite behaviour. The selection included two virgin hardwood cellulose grades (B600 and BC300), one pre-consumer recycled hardwood cellulose grade (PWC500, derived from industrial paper processing residues), one post-consumer recycled hardwood cellulose grade (ZZC500, recovered from post-consumer waste streams), and one secondary technical fibre grade (SIS250, recycled sisal from textile waste).

Comprehensive characterisation of the as-received fibres was performed prior to compounding. Morphological dimensions (length distribution, median length, thickness, and aspect ratio) were evaluated using Scanning Electron Microscopy (SEM, Carl Zeiss MERLIN, Oberkochen, Germany) and a GreCon 3D ParticleView analyser, Alfeld, Germany. Moisture content was determined according to ISO 18134-2 (oven-drying method at 105 °C), while bulk density was measured using standard volumetric compaction methods [24]. All fibres were utilized in their raw, as-received state without chemical surface pre-treatments; interfacial bonding with the hydrophobic rPP matrix was promoted via in situ compatibilization using 3 wt.% maleic anhydride-grafted polypropylene (MAPP, Licocene^®^ PP MA 7452 GR, Clariant, Muttenz, Switzerland). The physical, morphological, and historical attributes of the evaluated fibres are summarised in Table 1.

The numerical suffix associated with each material designation (e.g., 600 in B600 and 500 in ZZC500) represents the nominal average fibre length. All reinforcements were incorporated into the recycled polypropylene matrix at concentrations of 10%, 20% and 30% in order to evaluate the combined effects of fibre type, origin, and loading level on the resulting composite properties. This experimental design further enabled the determination of the most suitable reinforcement content for each fibre grade.

Commercial coupling agent Licocene^®^ PP MA 7452 GR (Clariant), a maleic anhydride-grafted polypropylene (MAPP), was used to enhance interfacial adhesion between the hydrophilic cellulose-based reinforcements and the hydrophobic polypropylene matrix. According to the findings of Bogataj et al. [25], MAPP content of 3 wt.% provides an optimal balance between interfacial compatibility, mechanical performance, and processability in recycled polypropylene composites reinforced with cellulose fibres, without compromising processability. Consequently, a constant MAPP concentration of 3 wt.% was incorporated into all composite formulations.

It is expected that this hybrid composition will contribute to the relatively high thermal conductivity of the reference rPP material (0.398 W/mK) compared with values typically reported for virgin polypropylene in the literature. The presence of inorganic fillers within the recycled matrix was confirmed by thermogravimetric analysis (TGA). In particular, the decomposition of calcium carbonate was identified above 600 °C, with the maximum decomposition rate occurring at approximately 722 °C, providing clear evidence of the mineral filler content in the recycled material.

### 2.2. Formulations and Manufacture of Composites

Table 2 presents the formulation matrix and coding system of the 16 materials evaluated in this work. The unreinforced recycled polypropylene (rPP) served as the reference material. The composite formulations were produced using recycled polypropylene as the matrix; 3 wt.% MAPP as the coupling agent; and the five cellulose-based reinforcements incorporated at concentrations of 10%, 20% and 30%. Each composite was identified according to the reinforcing type and fibre loading level. The complete formulation details are summarised in Table 2.

Following the development of the formulations, the composites were prepared through a controlled pre-mixing, compounding, and injection-moulding procedure. Particular attention was given to ensuring uniform fibre dispersion while minimising fibre degradation during processing, especially at higher reinforcement loadings.

Prior to compounding, the recycled polypropylene (rPP) matrix was sieved to obtain a uniform particle size distribution by removing oversized particles and excessive fines. The screened rPP, cellulose-based reinforcements, and MAPP coupling agent were then manually pre-mixed to achieve a homogeneous feed material.

Compounding was performed using a co-rotating twin-screw extruder (LAB-30, Useon, Nanjing, China) with a screw length-to-diameter ratio (L/D) of 44. Processing conditions were carefully optimised to adequate fibre dispersion and effective compatibilization while preventing thermal degradation of the cellulose reinforcements. The barrel temperature profile ranged from 30–60 °C in the feeding zone to 190–195 °C in the die zone. To minimise fibre attrition, the screw speed was adjusted according to the fibre loading level, with 150 rpm used for the 10 wt.% formulations and 100 rpm for the 20 and 30 wt.% formulations. Following extrusion, all materials, including the unreinforced rPP reference, were dried at 70 °C for 8 h prior to injection moulding.

Standard test specimens were subsequently produced using an injection moulding machine (Roboshot α-S50iA/330, FANUC, Yamanashi, Japan). Processing condition parameters were maintained constant for all formulations to ensure comparability of the results. The melt temperature was set to 180 °C and the mould temperature to 22 °C. Injection speed was maintained at 80 mm/s, and the screw rotation speed was 50 rpm. The total injection-cycle time was 40 s, including a cooling time of 20 s. A clamping force of 500 kN was applied during processing. All processing parameters were kept constant for all formulations to ensure comparability of the specimens.

The successful compounding and injection moulding of these composite formulations at the laboratory scale demonstrate promising thermo-mechanical processability. While these results highlight the potential for upcycling secondary cellulosic fibres into rPP matrices, full industrial scalability, fibre length retention during repeated processing, and batch-to-batch reproducibility require further systematic validation in commercial manufacturing environments.

### 2.3. Thermal Characterisation of the Composites

A comprehensive thermal characterisation was conducted to investigate the influence of reinforcement type and loading level on the thermal behaviour of the recycled polypropylene composites. The analysis included thermogravimetric analysis (TGA) to evaluate thermal stability, differential scanning calorimetry (DSC) to assess phase-transition behaviour and crystallinity, thermodilatometric analysis (DIL) to determine dimensional stability, and thermal conductivity measurements to evaluate heat-transfer performance. All analyses were performed on the 16 formulations developed in this study.

Thermogravimetric analysis (TGA) and differential scanning calorimetry (DSC) were employed to evaluate the thermal behaviour of the developed composites. TGA was used to determine the onset degradation temperature (T_onD_) and assess thermo-oxidative stability, whereas DSC was applied to characterise melting behaviour and crystallinity, including the onset melting temperature (T_onM_), peak melting temperature (T_M_), and degree of crystallinity (Xc).

The thermal behaviour of the composites was evaluated using a simultaneous TGA-DSC thermal analyser (SDT Q600, TA Instruments, New Castle, DE, USA). Measurements were conducted in a single continuous heating scan from room temperature (25 °C) to 1000 °C at a constant heating rate of 10 °C/min under an air atmosphere with a flow rate of 30 mL/min. Samples weighing between 10 and 20 mg were analysed in 70 µL alumina crucibles. The single heating cycle was selected to record thermal transitions, melting characteristics, and matrix crystallinity directly resulting from the compounding and injection moulding processing history. Temperature and heat flow calibrations were performed using high-purity Indium and sapphire standards.

The degree of crystallinity was calculated using Equation (1):

(1) $$\mathrm{Xc}=\frac{∆H_{M}}{∆H_{M}^{0}\cdot W_{\mathrm{rPP}}}\times 100$$

where ∆H_M_ is the measured melting enthalpy of the composite (J/g), $∆H_{M}^{0}$ is the theoretical melting enthalpy of 100% crystalline polypropylene (209 J/g), and W_rPP_ is the mass fraction of the as-received recycled polypropylene matrix in the composite formulation (W_rPP_ = 1 − W_fibre_ − W_MAPP_). Here, Xc reflects the effective crystallinity relative to the total industrial rPP matrix batch (inclusive of its intrinsic mineral fillers, ~21.5–30 wt.% talc/CaCO_3_, as confirmed by TGA ash residue), providing a consistent baseline to evaluate the relative nucleating effect of cellulosic fibres across all composite formulations [26,27].

Thermodilatometric analysis (DIL) was employed to investigate the dimensional response of the composites to temperature variations and to assess their thermal dimensional stability. Measurements were performed using a horizontal dilatometer (DIL 402C, Netzsch, Selb, Germany) from room temperature (25 °C) to 160 °C at a constant heating rate of 2 °C/min under a nitrogen (N_2_) atmosphere (n = 3 replicates per formulation). From the resulting expansion curves, the maximum relative expansion (dL/L_0_)max and the coefficient of linear thermal expansion (α_40–120_*)* were determined.

The coefficient of linear thermal expansion was calculated using Equation (2):

(2) $$\alpha_{40-120}=\frac{{\left(\frac{\mathrm{dL}}{\mathrm{Lo}}\right)}_{T2}-{\left(\frac{\mathrm{dL}}{\mathrm{Lo}}\right)}_{T1}}{T_{2}-T_{1}}\times 10^{6}$$

where ${\left(\mathrm{dL}/L_{0}\right)}_{T_{1}}$ and ${\left(\mathrm{dL}/L_{0}\right)}_{T_{2}}$ correspond to the relative dimensional changes at 40 °C and 120 °C, respectively, across a temperature interval of ΔT = 80 °C.

The temperature interval between 40 °C and 120 °C was selected to avoid the influence of secondary relaxation phenomena at low temperatures and melting-related dimensional changes occurring at temperatures above 120 °C. Thermal conductivity and thermal diffusivity were determined to assess the heat-transfer behaviour of the composites and their suitability for applications where thermal management and dimensional stability are of importance. Measurements were carried out using the (MTPS) method and a transient heat flux analyser (Trident, C-Therm Technologies, Fredericton, NB, Canada) in accordance with ASTM D7984. The MTPS technique enables rapid and reliable determination of thermal transport properties through transient heat-flow measurements at the specimen surface. Flat specimens measuring 50 × 50 × 4 mm^3^ were tested at room temperature under transient heat flux conditions (n = 5 measurements per formulation), reporting Mean ± SD and Relative Standard Deviation (RSD %).

### 2.4. Mechanical Characterisation

Tensile testing was conducted in accordance with ISO 527-1 and ISO 527-2 standards using type 1A dog-bone specimens (150 × 10 × 4 mm^3^) on a Zwick/Roell universal testing machine equipped with a 10 kN load cell [28,29]. Tests were performed at room temperature (23 ± 2 °C) at a crosshead speed of 1 mm/min for determination of Young’s Modulus (Et) and 50 mm/min for evaluation of Tensile Strength (σ_M_) and Elongation at Break (ɛ_t_). A total of n = 10 specimens per composite formulation were tested.

Charpy impact testing was performed according to DIN EN ISO 179-1/DIN 53453 (unnotched, edgewise orientation) using a calibrated 5 J pendulum hammer [30,31]. Rectangular specimens measuring 50 × 10 × 4 mm^3^ were struck horizontally in a single rapid impact. The absorbed impact energy was normalized to the cross-sectional area and reported in kJ/m^2^. A minimum of n = 6 specimens per formulation were evaluated to ensure statistical reliability.

### 2.5. Morphological and Microstructural Characterisation (SEM)

Scanning Electron Microscopy (SEM) was employed to evaluate the distribution, morphological characteristics, and interfacial bonding of the reinforcing fibres and fillers within the polymer matrix. The morphological analysis of fractured composite samples was conducted using a Quanta 250 SEM (FEI Company, Eindhoven, The Netherlands). Prior to imaging, cross-sections of the specimens fractured during tensile testing were mounted onto SEM sample stubs using conductive adhesive tape and sputter-coated with a thin layer of gold to enhance conductivity and prevent electrostatic charging. Imaging was conducted under high-vacuum mode with an accelerating voltage optimized for polymeric materials (between 10 and 20 kV). Micrographs were collected at magnifications of 100×, 500×, and 1000× to evaluate fibre dispersion, filler alignment, and the presence of any structural defects such as agglomerates or voids.

### 2.6. Statistical Analysis

Data from tensile (n = 10), unnotched Charpy impact (n = 6), and thermal conductivity/effusivity (n = 5 transient pulse measurements) tests are presented as Mean ± Standard Deviation (Mean ± SD). For thermal conductivity (λ) and effusivity (ε), the relative standard deviation (RSD %) was maintained below 1.0% across all formulations, demonstrating high measurement repeatability. One-Way Analysis of Variance (ANOVA) followed by Tukey’s HSD post-hoc test was performed at a significance level of *p* < 0.05 to evaluate statistically significant differences among composite formulations and the reference rPP matrix.

## 3. Results and Discussion

**Figure 1:**
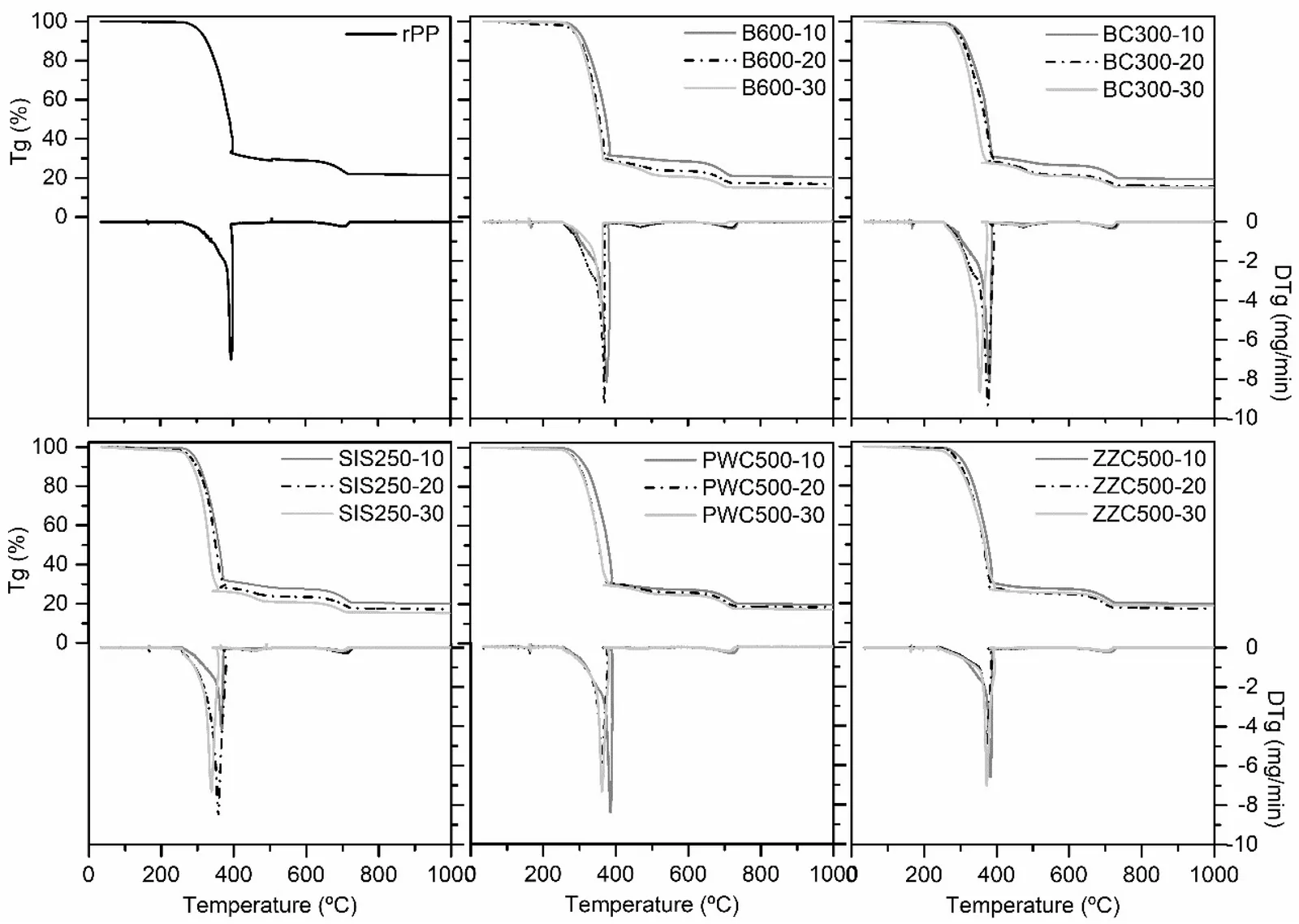
Thermogravimetric analyses of the polymer matrix and composites reinforced with different fibres.

Figure 1 presents the thermogravimetric (TG) and derivative thermogravimetric (DTG) curves of the reference recycled polypropylene (rPP) matrix and the composites reinforced with different cellulose-based fibres. The thermogravimetric behaviour of the reference rPP reveals a negligible mass loss of approximately 0.3% up to 225 °C, which can be attributed to the evaporation of residual moisture.

The onset of thermal degradation (T_onD_) occurs at 274 °C, marking the beginning of polymer chain scission and the release of volatile degradation products. The primary degradation stage extends from T_onD_ to approximately 400 °C, during which time the material undergoes a mass loss of 67.2%. The maximum degradation rate is observed at 379 °C, as indicated by the DTG curve in Figure 1.

Beyond 400 °C, the TG curve exhibits a relatively stable region characterised by a substantially lower rate of mass loss, indicating that most organic constituents have already decomposed and that the remaining residue is primarily inorganic in nature. A second degradation stage becomes evident above 600 °C and is associated with the decomposition of calcium carbonate present as a mineral filler in the recycled polypropylene matrix. This process reaches its maximum decomposition rate at approximately 722 °C, as shown by the DTG curve. The cumulative mass loss between 400 °C and the final test temperature amounts to 11.0%.

Overall, the thermogravimetric profile of the reference rPP is characteristic of a mineral-filled polypropylene compound, exhibiting good thermal stability up to T_onD_ and a distinct high-temperature decomposition stage associated with the presence of calcium carbonate fillers.

As summarised in Table 3, the incorporation of cellulose-based reinforcements resulted in a systematic reduction in the onset degradation temperature (T_onD_) relative to the reference rPP matrix, indicating a moderate decrease in thermo-oxidative stability. This behaviour is commonly reported for natural fibre-reinforced thermoplastic composites and is primarily associated with the lower thermal resistance of lignocellulosic constituents compared with the polypropylene matrix [32,33].

Depending on the reinforcing type and loading level, T_onD_ decreased by 2.6–5.1%, with the lowest value recorded for the ZZC500-30 composite. The progressive reduction in thermal stability can be attributed to the increasing proportion of fibre constituents that undergo thermal degradation prior to the onset of polypropylene decomposition, as well as to fibre–matrix interfacial effects that facilitate the initiation of degradation processes. Despite these reductions, the thermal stability of all formulations remained adequate for conventional melt-processing operations.

**Figure 2:**
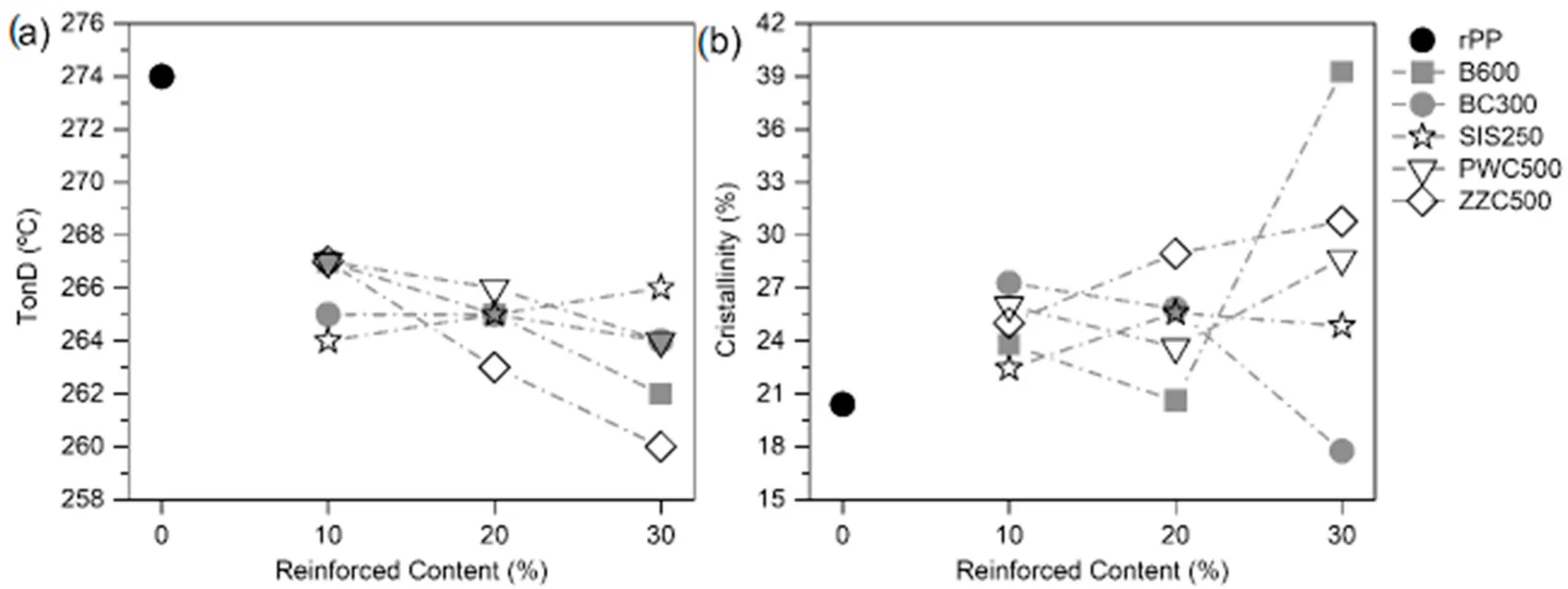
Influence of reinforcing material content on composite thermal and structural properties: (**a**) Thermal degradation onset temperature and (**b**) Degree of crystallinity.

Figure 2a illustrates the influence of reinforcement content on the onset degradation temperature (T_onD_) for the different fibre types. The effect of increasing fibre loading was found to depend strongly on the origin and type of reinforcement.

For composites reinforced with B600, PWC500 and ZZC500 fibres, a progressive decrease in T_onD_ was observed as the fibre content increased from 10 to 30 wt.%. The most pronounced reduction occurred in the ZZC500 series, where T_onD_ decreased from 267 °C to 260 °C, followed by the B600 composites, which exhibited a decrease from 267 °C to 262 °C. In contrast, the PWC500 series showed a less significant reduction, with T_onD_ decreasing only from 267 °C to 264 °C over the same reinforcement range. A different trend was observed for the SIS250-reinforced composites. Increasing the fibre content from 10% to 30 wt.% resulted in a slight increase in T_onD_, from 264 °C to 266 °C, indicating a modest improvement in thermo-oxidative stability. Consequently, the difference between the composite and the reference rPP matrix (T_onD_ = 274 °C) became less pronounced at higher SIS250 loadings.

In the case of the BC300 series, the onset degradation temperature remained essentially unaffected by fibre content, with T_onD_ values remaining stable at approximately 265 °C across all reinforcement levels. These results suggest that the influence of reinforcement loading on thermal stability is highly dependent on fibre origin and composition.

With respect to mass-loss behaviour, only minor differences were observed among the investigated formulations (Table 3). Increasing the fibre content from 10 to 30 wt.% resulted in a modest increase in mass loss during the primary degradation stage (225–400 °C) and a corresponding reduction in the final residue content. The most pronounced changes were observed for the SIS250 and B600 series, whereas the PWC500 and ZZC500 composites exhibited comparatively limited variations. Overall, these results indicate that the incorporation of cellulose-based reinforcements has only a limited influence on the degradation mechanism of the composites. While fibre type and loading level affect the onset degradation temperature, their impact on the subsequent degradation stages and residual mass is relatively small, suggesting that the thermal decomposition behaviour remains predominantly governed by the recycled polypropylene matrix [32].

**Figure 3:**
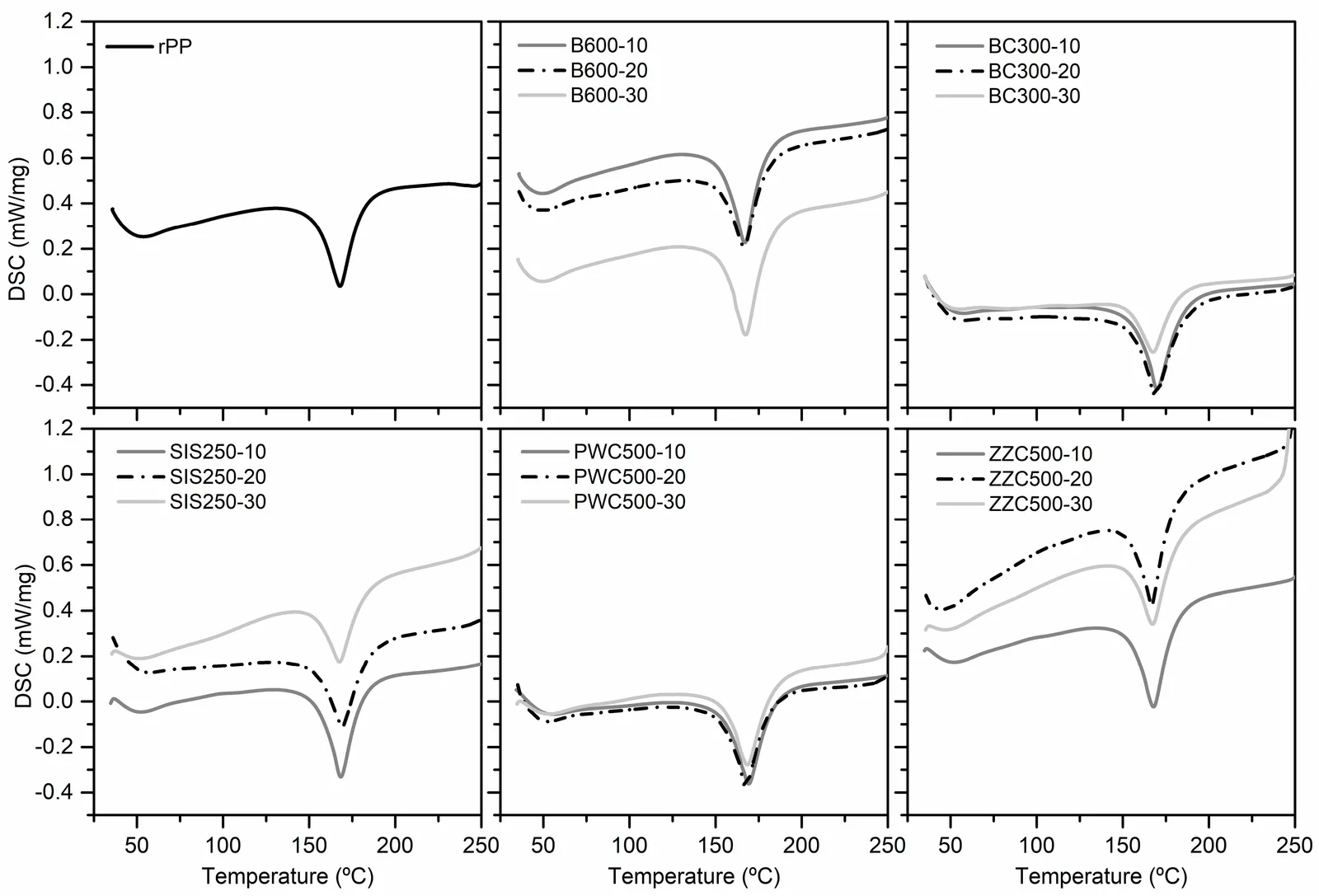
Differential scanning calorimetry of the polymer matrix and composites reinforced with different fibres.

Figure 3 shows the DSC thermograms of the reference rPP matrix and the corresponding fibre-reinforced composites. All materials exhibited a characteristic endothermic melting peak associated with polypropylene melting. For the reference matrix, the onset melting temperature (T_onM_) was measured at 153 °C, while the maximum melting temperature (T_M_) occurred at 168 °C. The results presented in Table 4 demonstrate that neither fibre origin (virgin, pre-consumer recycled, or post-consumer recycled) nor fibre loading affected the melting characteristics of the composites. The limited variation in T_onM_ and T_M_ among the formulations suggests that the incorporation of cellulose-based reinforcements had little influence on the crystal structure and melting behaviour of the recycled polypropylene matrix.

The degree of crystallinity (Xc) was determined from the melting enthalpy (ΔH_M_) derived from the DSC thermograms, assuming a melting enthalpy of 209 J/g for fully crystalline polypropylene [26,27]. The resulting crystallinity values are summarised in Table 4.

The reference rPP matrix exhibited a crystallinity of 20.4%. For most formulations, the incorporation of cellulose-based reinforcements resulted in an increase in crystallinity, suggesting that the fibres acted as nucleating sites during crystallisation of the polypropylene matrix. The only exception was the BC300-30 composite, for which the crystallinity decreased to 17.8%, representing a 12.9% reduction relative to the reference material.

The baseline crystallinity of the unreinforced post-industrial rPP matrix (Xc = 20.4%) is influenced by its intrinsic mineral filler content (~21.5–30 wt.% talc and CaCO_3_, confirmed by TGA ash residue), where platy minerals like talc act as efficient heterogeneous nucleating agents. Upon the addition of cellulosic fibres, a further enhancement in Xc was observed across most formulations, reaching a maximum of 39.3% in short-fibre composite B600_30. This substantial increase is attributed to the high specific surface area and elevated particle density of the short B600 fibres, which provide abundant transcrystallization sites for PP crystal growth. Since all composite formulations contained a fixed concentration of 3 wt.% MAPP, the observed increase in matrix crystallinity represents the combined contribution of cellulosic fibres acting as heterogeneous nucleating agents and MAPP facilitating interfacial wetting. While literature reports suggest that MAPP enhances crystal growth by improving stress transfer and interfacial nucleation, the relative contribution of each component cannot be isolated in the absence of an uncompatibilized (MAPP-free) control [34].

Conversely, the BC300 series displayed a reduction in crystallinity with increasing fibre content. This atypical behaviour suggests that, beyond a critical fibre concentration, the nucleating efficiency of BC300 fibres may be compromised. Such behaviour could be associated with fibre agglomeration, restricted polymer chain mobility, or reduced interfacial effectiveness, all of which may inhibit crystal formation and growth.

**Figure 4:**
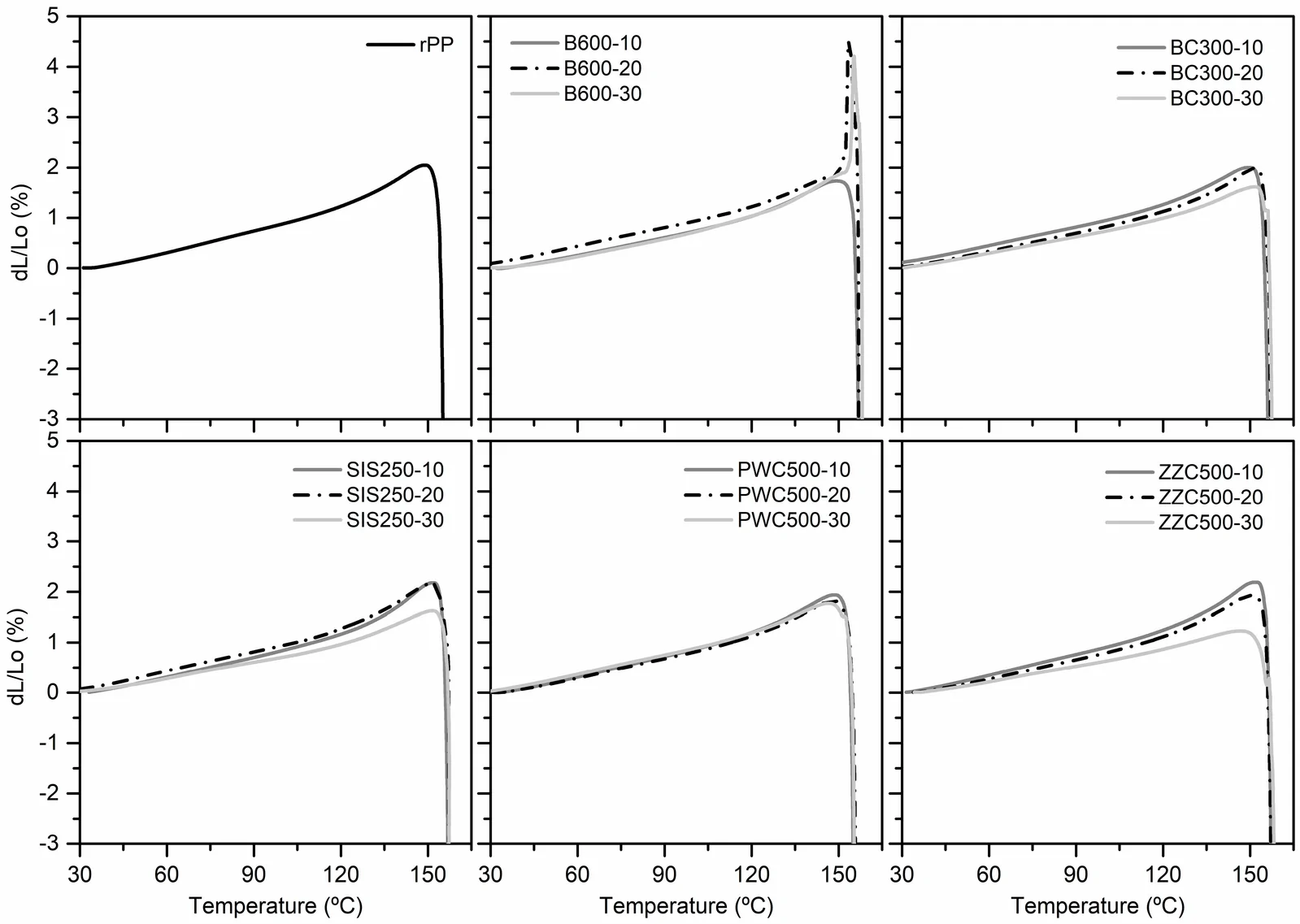
Thermodilatometric analysis of the polymer matrix and composites reinforced with different fibres.

Figure 4 shows the thermodilatometric response of the reference rPP matrix and the corresponding fibre-reinforced composites. For all materials, the relative dimensional change increased continuously with temperature, reaching a maximum value at approximately 150 °C. This temperature corresponds closely to the onset of melting observed in the DSC measurements, beyond which the polymer matrix loses its structural rigidity and undergoes a marked reduction in dimensional stability.

While the addition of cellulosic fibres consistently reduced the coefficient of linear thermal expansion (α_40–120_) across all composite families in the solid-state service range (40–120 °C), the maximum relative thermal expansion (dL/L_0,max_) measured near the softening transition (~150 °C) showed family-dependent variations. Specifically, formulations B600-20 (4.48%) and B600-30 (4.21%) exhibited higher maximum expansion than the reference rPP (2.04%), which can be attributed to localized fibre agglomeration and stress relief prior to melting.

Table 5 summarises the coefficient of linear thermal expansion (α_40–120_) for the reference rPP matrix and the fibre-reinforced composites. The incorporation of cellulose-based reinforcements resulted in a reduction in α_40–120_ for all formulations, with decreases ranging from 1.5% to 28.9% relative to the unreinforced matrix. The greatest reduction was observed for the ZZC500-30 composite, which exhibited a 28.9% lower coefficient of thermal expansion than the reference rPP.

**Figure 5:**
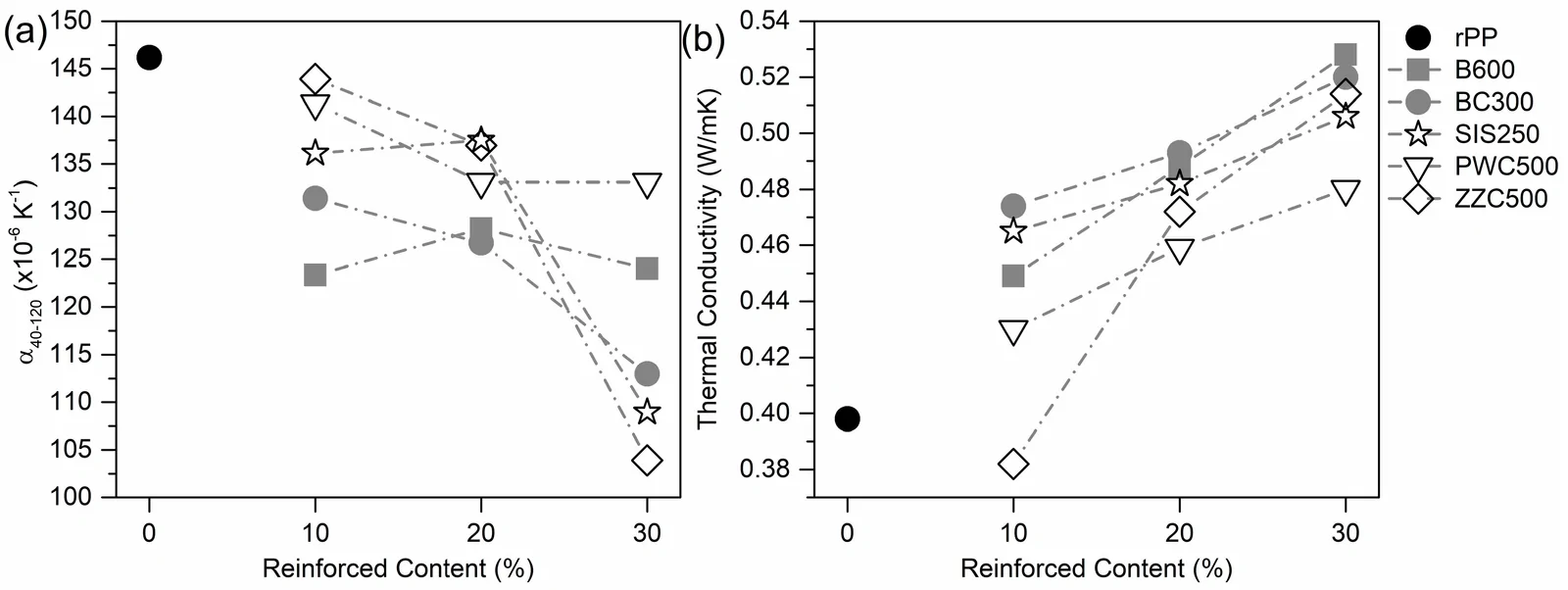
Thermal properties of composites as a function of reinforcing material content: (**a**) linear thermal expansion; (**b**) thermal conductivity.

The influence of reinforcement content on α_40–120_ depended on the fibre type. For the B600 and PWC500 series, the reduction remained relatively constant with increasing fibre loading, averaging approximately 15% and 9%, respectively. In contrast, the BC300, SIS250, and ZZC500 series exhibited a progressive decrease in α_40–120_ as the reinforcement content increased. This effect became particularly pronounced at a fibre loading of 30 wt.%, with the ZZC500-30 and SIS250-30 composites showing the largest reductions relative to their corresponding 20 wt.% formulations (Figure 5a).

The observed reduction in thermal expansion can be attributed to the constraining effect of the cellulose-based reinforcements on the polymer matrix. Fibres typically exhibit lower coefficients of thermal expansion and higher stiffness than polypropylene, thereby restricting polymer chain mobility and reducing thermally induced deformation [9,35,36]. This effect is further enhanced by strong fibre–matrix interfacial adhesion, which improves stress transfer between the phases and enables the reinforcement to more effectively limit matrix expansion [37]. The presence of MAPP likely contributed to this behaviour by promoting interfacial compatibility between the hydrophilic fibres and the hydrophobic polypropylene matrix. Since the baseline inorganic mineral filler content (~30 wt.%) inherent to the recycled rPP matrix remained constant across all blends, the additional 28.9% reduction in α_40−120_ and the 32.7% increase in thermal conductivity are exclusively attributed to the synergistic effect of the ZZC500 cellulosic fibres combined with the MAPP compatibiliser.

The observed reduction in the coefficient of linear thermal expansion translates directly into enhanced dimensional stability, particularly at higher fibre loadings. From an engineering perspective, this behaviour is highly desirable, as it reduces the susceptibility of polymer components to thermally induced deformation, residual stresses, and dimensional mismatch during service.

As a result, fibre-reinforced recycled polypropylene composites may offer significant advantages in applications requiring dimensional precision under moderate thermal cycling conditions. Such applications include moulded technical parts, packaging components, household appliances, and other non-structural products where thermal dimensional stability is a critical performance criterion [38,39,40,41,42].

The thermal conductivity values of the reference rPP matrix and the developed composites are summarised in Table 4. With the exception of the ZZC500-10 formulation, all fibre-reinforced composites exhibited higher thermal conductivity than the reference rPP matrix (0.398 W/mK). The highest thermal conductivity was recorded for the B600-30 composite (0.528 W/mK), corresponding to an increase of 32.7% relative to the unreinforced matrix. In contrast, the ZZC500-10 composite exhibited a thermal conductivity of 0.382 W/mK, representing a decrease of 4.0%.

It should be noted that the thermal conductivity of the reference rPP matrix was higher than values typically reported for virgin polypropylene in the literature [21]. This behaviour can be attributed to the presence of approximately 30 wt.% mineral fillers, primarily talc and calcium carbonate, already contained within the recycled polypropylene matrix.

As shown in Figure 5b, thermal conductivity generally increased with increasing fibre content. This behaviour can be attributed to the introduction of additional heat-transfer pathways within the composite structure, as well as to changes in composite density and morphology resulting from fibre incorporation. The most pronounced increases were observed for the B600 and ZZC500 series. For example, thermal conductivity increased from 0.449 to 0.528 W/mK for the B600 composites and from 0.382 to 0.514 W/mK for the ZZC500 composites as the fibre content increased from 10 to 30 wt.%, corresponding to increases of 17.6% and 34.6%, respectively. In contrast, the BC300, SIS250, and PWC500 series exhibited more moderate increases of approximately 10% over the same reinforcement range.

**Figure 6:**
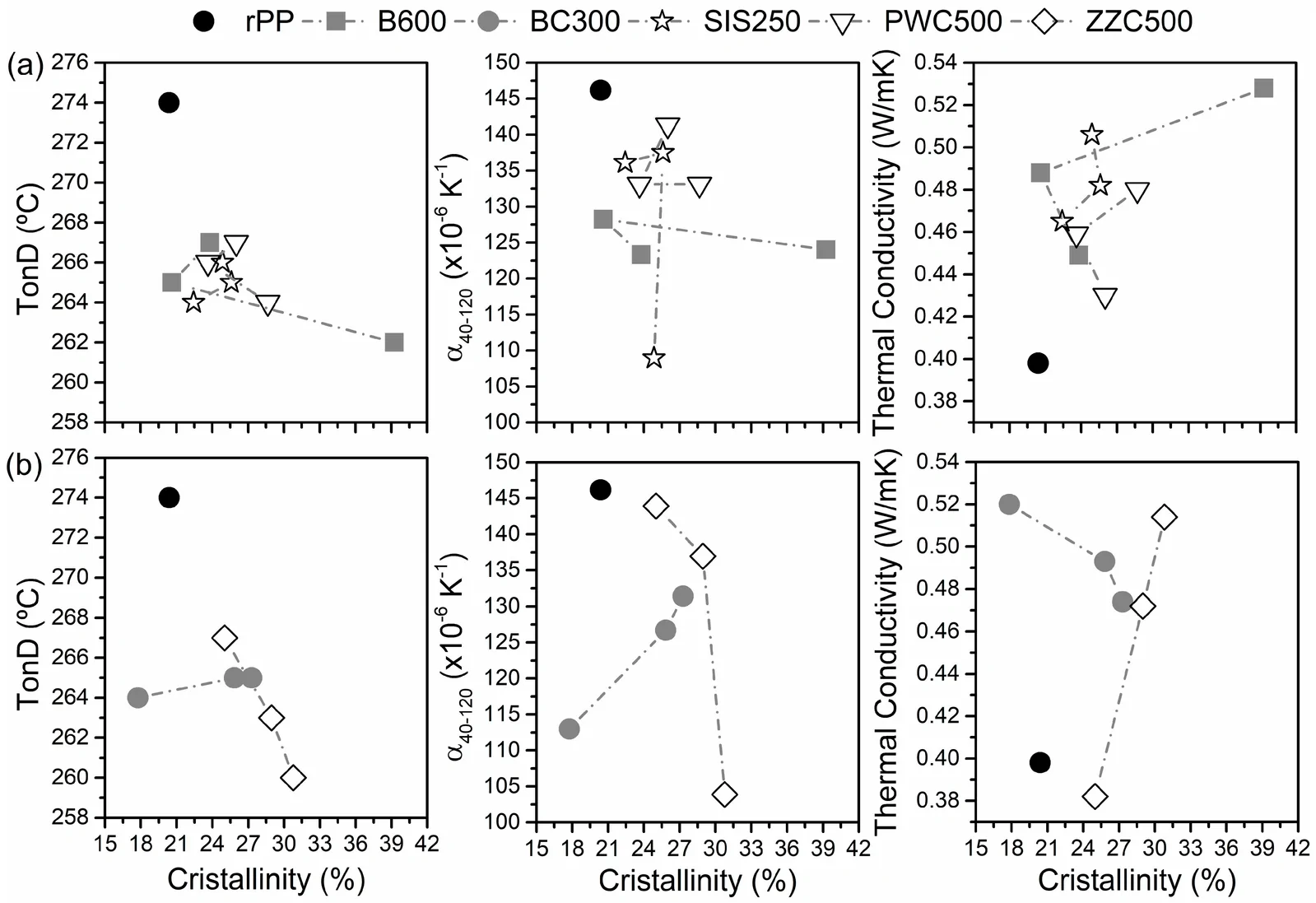
Relationship between crystallinity and the thermal properties of composites reinforced with different fibres. (**a**) B600, SIS250, and PWC500 (**b**) BC300 and ZZC500.

To further investigate the relationship between thermal properties and microstructural development, the thermal conductivity and dimensional stability results were analysed in conjunction with the crystallinity data. For the B600, SIS250, and PWC500 series, no clear correlation was observed between crystallinity and the measured thermal properties (Figure 6a). This suggests that factors such as fibre morphology, fibre distribution, interfacial characteristics, and the intrinsic thermal properties of the reinforcement may have a greater influence on composite behaviour than crystallinity alone.

In contrast, the BC300 and ZZC500 series exhibited a more systematic relationship between crystallinity and thermal performance (Figure 6b). However, opposite trends were observed. Increasing BC300 content resulted in a progressive decrease in crystallinity, whereas increasing ZZC500 content promoted crystallisation of the polypropylene matrix. These results indicate that the influence of cellulose-based reinforcements on crystallisation behaviour is strongly dependent on fibre origin and morphology.

Thermal conductivity increased progressively with fibre loading across all composite families, reaching its maximum absolute value in short-fibre composite B600-30 (0.528 W/m·K, representing a 32.7% enhancement over the reference rPP matrix), closely followed by BC300-30 (0.520 W/m·K, +30.7%). This response is attributed to the higher particle-number density and effective interfacial contact area of shorter B600 fibres, which create efficient thermal conduction pathways within the matrix. However, this advantage was accompanied by lower crystallinity and higher coefficients of thermal expansion at elevated fibre loadings, indicating reduced dimensional stability. In contrast, the ZZC500 composites combined enhanced crystallinity with lower thermal expansion coefficients, resulting in superior dimensional stability while maintaining relatively high thermal conductivity. From an application perspective, these findings suggest that both BC300- and ZZC500-reinforced composites may be suitable for components requiring efficient heat transfer, although the ZZC500 formulations offer a more favourable balance between thermal conductivity and dimensional stability.

The progressive increase in crystallinity observed for most formulations, particularly in the PWC500 and ZZC500 series, suggests the development of a more ordered polymer structure, which is commonly associated with enhanced stiffness and improved load-bearing capability. Previous studies by Bhardwaj et al. [9] have shown that cellulose fibre-reinforced recycled polymer composites can achieve mechanical performance comparable to that of virgin polymers systems when adequate fibre dispersion and fibre–matrix compatibility are achieved. In the present study, the nucleating effect of the cellulose-based fibres, combined with the coupling action of the 3 wt.% MAPP agent, promoted increased crystallinity and improved interfacial interactions within the composite structure. These microstructural changes are expected to contribute positively to the mechanical performance of the developed materials; however, confirmation of this effect requires dedicated mechanical characterisation, including tensile, flexural, and impact testing.

The tensile and impact characterisation results of the circular composites and the reference rPP matrix are summarised in Table 6 (reported as Mean ± Standard Deviation). The tensile test results clearly demonstrate the substantial reinforcing effect of the cellulosic fibres within the rPP matrix. The Young’s modulus (Et) values show a directly proportional increase with higher fibre content across all composite families, indicating enhanced stiffness. The reference rPP matrix exhibited an initial Young’s modulus of 2414 ± 60 MPa. This value increased up to 3492 ± 58 MPa for the composite with 30 wt.% of recycled sisal fibres (SIS250_30), representing an increase of 45%. Substantial stiffness improvements were also observed for the virgin cellulose families (BC300_30 at 3363 ± 65 MPa, +39%) and post-consumer recycled cellulose (ZZC500_30 at 3166 ± 112 MPa, +31%), all showing statistically significant differences compared to the neat matrix (*p* < 0.05, One-Way ANOVA and Tukey’s test).

Similarly, tensile strength (σ_M_) improved markedly with fibre loading (*p* < 0.05). Tensile strength (σ_M_) improved across composite formulations, though the extent of reinforcement varied significantly depending on the fibre type and loading level. Compared to the reference rPP matrix (21.8 ± 0.8 MPa), tensile strength gains ranged from +12.8% in B600_20 (24.6 ± 0.2 MPa) up to +91.7% in SIS250_30 (41.8 ± 0.8 MPa). For the remaining composite families (BC300, ZZC500, and PWC500, as well as B600_10 and B600_30), tensile strength improvements fell between +21% and +43% (reaching values between 26.3 y 31.2 MPa). In contrast, the elongation at break (ε_t_) systematically decreased as fibre content rose. For example, rPP dropped from 3.7 ± 0.9% to 1.8 ± 0.1% in B600_30 and 2.0 ± 0.2% in BC300_30. This reduction is a classic trade-off in composite materials, where the increased rigidity of the reinforcing fibres restricts the localized deformation of the polymer chains under tensile stress.

The unnotched Charpy impact strength results are also detailed in Table 6. A general improvement in impact resistance was recorded for most composite formulations compared to the unreinforced rPP matrix (10.6 ± 0.5 kJ/m). Notably, the B600_10 formulation exhibited an impact strength of 15.6 ± 1.1 kJ/m^2^ (+47% increase, *p* < 0.05). However, exceptions to this trend were observed at high loadings. Specifically, B600_20 (9.0 ± 2.2 kJ/m^2^) and B600_30 (8.9 ± 0.7 kJ/m^2^) showed a decrease in impact toughness (without statistically significant variation from the reference matrix, *p* > 0.05). This deviation is likely associated with fibre agglomeration at higher weight fractions, which acts as a stress concentrator and promotes premature cracks. Additionally, composites with ZZC500 fibres at 10 wt.% (ZZC500_10 at 9.8 ± 2.7 kJ/m^2^) exhibited a slight decrease in toughness due to the lower quality and shorter structural integrity typical of recycled post-consumer cellulose sources.

**Figure 7:**
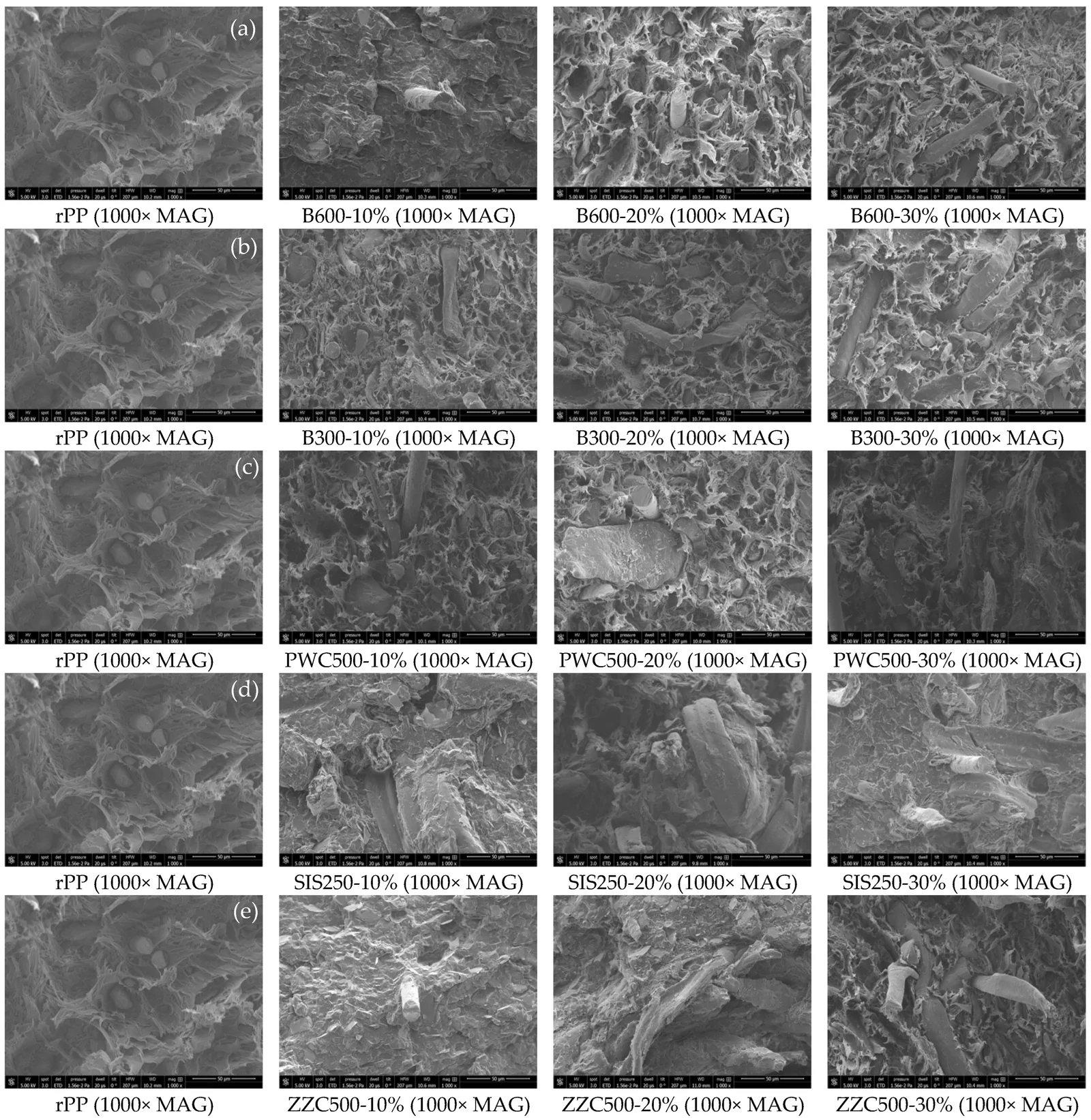
SEM micrographs (1000× magnification) of the tensile-fractured cross-sections of the reference rPP matrix and (**a**) B600_20%, (**b**) BC300_20%, (**c**) PWC500_20%, (**d**) ZZC500_20%, and (**e**) SIS250_20% composites.

SEM micrographs of tensile-fractured cross-sections (Figure 7) revealed regular, cylindrically shaped fibres with uniform circular cross-sections randomly dispersed throughout the rPP matrix. This morphology confirms the presence of minor residual glass fibre traces incorporated incidentally into the sorted post-industrial regrind stream during industrial waste processing. Rather than causing structural defects, these residual fibres appear well-embedded within the compatibilized matrix, contributing synergistically to matrix rigidity alongside the intentionally added cellulosic fibres.

Crucially, SEM micrographs of tensile-fractured surfaces revealed good fibre dispersion, with an absence of extensive fibre pull-out or prominent interfacial perimeter voids around the cellulosic reinforcements. While this morphology indicates robust interfacial adhesion and matrix wetting—behaviour typically associated with MAPP compatibilization in natural fibre/polypropylene systems—this interpretation is presented cautiously as a descriptive morphological feature, given that an uncompatibilized control was not evaluated [28,31]. In particular, formulations containing PWC500 and B600 fibres demonstrated outstanding interfacial bonding at all fibre loading levels, indicating consistent compatibility. Similarly, composites with ZZC500 fibres at 30% loading and SIS250 fibres at higher percentages exhibited highly favourable interfacial compatibility. The structural integrity and lack of morphological defects confirmed by SEM correlate perfectly with the enhanced tensile and impact properties of these circular materials.

## 4. Conclusions

This study successfully demonstrates the technical viability of manufacturing circular composite materials with high dimensional and functional stability by upcycling post-industrial recycled polypropylene (rPP) derived from household appliance manufacturing and reinforcing it with various cellulosic fibres. The rPP matrix utilized in this study contains an intrinsic mineral filler content of approximately 30 wt.% (talc and calcium carbonate) originating from its previous lifecycle. By incorporating 3 wt.% maleic anhydride-grafted polypropylene (MAPP) as a compatibilizer, the thermodynamic interfacial incompatibility between the non-polar matrix and the polar cellulose reinforcements was effectively resolved. The systematic evaluation of these composite systems yields the following key conclusions:

- Morphological and Microstructural Analysis (SEM): Scanning Electron Microscopy (SEM) micrographs of the tensile-fractured cross-sections confirmed excellent dispersion of the cellulosic fibres and robust interfacial adhesion across almost all analysed formulations. The widespread absence of fibre pull-out or perimeter voids at the fibre–matrix boundary indicates favourable interfacial wetting and mechanical interlocking. While this morphological feature is consistent with MAPP compatibilization reported in the literature, it is interpreted as a descriptive microstructural state rather than direct chemical proof, as an uncompatibilized baseline was not evaluated.
- Mechanical Properties (Tensile and Impact): Mechanical characterisation demonstrated a clear reinforcing effect directly proportional to fibre loading: Composite stiffness increased systematically with fibre content; the Young’s modulus (Et) of the reference rPP matrix (2414 MPa) rose by 45% for the composite reinforced with 30 wt.% recycled sisal (SIS250_30, 3492 MPa), followed by virgin cellulose (BC300_30, 3363 MPa) and post-consumer recycled cellulose (ZZC500_30, 3166 MPa). Tensile strength (σM) improved across composite formulations, ranging from +12.8% in B600_20 up to +91.7% in SIS250_30 (with most formulations showing gains between +21% and +43%). Conversely, elongation at break (ɛt) systematically decreased due to the increased rigidity and restricted polymer chain mobility introduced by the cellulose fibres. Unnotched Charpy impact strength of the rPP matrix (10.6 kJ/m^2^) improved significantly at low fibre loadings, peaking at 15.6 kJ/m^2^ (+47%) for the 10 wt.% virgin long wood fibre composite (B600_10). However, at high loadings (20 and 30 wt.%), the formation of local fibre agglomerates led to a critical drop in impact toughness (down to 8.9 kJ/m^2^ for B600_30) due to their action as stress concentrators. The degraded post-consumer recycled fibres with shorter lengths (ZZC500_10) also exhibited a slight drop at low dosage (9.8 kJ/m^2^) due to their lower structural integrity.
- Thermal Stability (TGA): Thermogravimetric curves revealed that the incorporation of cellulosic fibres leads to a minor reduction in the onset degradation temperature (TonD), ranging from 2.6% to 5.1% compared to the reference rPP matrix (274 °C). This behaviour is attributed to the lower intrinsic thermal stability of the lignocellulosic components relative to the synthetic polymer. The lowest onset degradation temperature was recorded for the ZZC500_30 composite (260 °C), followed closely by B600_30 (262 °C). Nonetheless, all formulations maintained a highly secure thermal processing window well above 200 °C. At temperatures above 600 °C, the decomposition of residual calcium carbonate in the rPP matrix was observed, reaching its maximum degradation rate at approximately 722 °C.
- Phase Transitions and Crystallization Behaviour (DSC): Differential Scanning Calorimetry (DSC) analysis showed that the main thermal transitions of the matrix onset melting temperature (T_onM_ of approx. 153 °C) and peak melting temperature (TM of approx. 168 °C) remained unaffected across all formulations, confirming that the crystal structure of the polypropylene is not morphologically altered. However, the matrix crystallinity (Xc) (20.4%) increased significantly in nearly all composites due to the heterogeneous nucleating effect of the cellulose fibres and MAPP coupling. The most prominent crystallization increase occurred in the B600_30 composite, reaching 39.3% crystallinity. As an exception, the virgin short wood fibre series (BC300) showed a decrease in crystallinity at maximum loading (17.8% in BC300_30), which indicates a steric effect that restricts the molecular mobility of polymer chains when a critical concentration of short fibres is reached.
- Dimensional Stability (DIL): Thermodilatometric measurements over the solid-state service interval (40 °C to 120 °C) proved that cellulosic fibres systematically reduced the coefficient of linear thermal expansion (α_40–120_) across all composite formulations, achieving a maximum reduction of 28.9% in ZZC500-30 (104 K^−1^ vs. 146 K^−1^ for rPP). In contrast, the maximum relative thermal expansion (dL/L_0,max_) near the softening transition (~150 °C) varied by family, showing higher values in B600-20/30 due to localized stress relaxation prior to melting. Thus, dimensional stabilization by cellulosic fibres is strictly effective in the solid-state operational window.
- Heat-Transfer Performance (MTPS): Thermal conductivity (λ) increased in a directly proportional manner with fibre loading due to the creation of more efficient heat-transfer pathways within the composite structure. The baseline conductivity of the reference rPP matrix was already high (0.398 W/mK) due to the talc and calcium carbonate mineral fillers pre-existing in the recycled source. However, conductivity peaked in the B600_30 composite at 0.528 W/mK (+32.7%), followed by the ZZC500_30 formulation at 0.514 W/mK (+29.1%). Both series are highlighted as highly suitable for technical components requiring thermal dissipation and dimensional shape retention. Optimal Composite Selection.

By correlating the complete set of test results, it can be concluded that the B600_10 composite (10 wt.% virgin long wood cellulose, 3 wt.% MAPP) represents the optimal candidate for applications requiring high ductility and toughness, as it successfully increases tensile strength by 32% (28.7 MPa) and maximizes Charpy impact toughness by 47% (15.6 kJ/m^2^) while maintaining favourable thermal properties and dispersion, and the ZZC500_20 composite (20 wt.% post-consumer recycled cellulose) represents the ultimate circular solution, as it enables the development of high-performance composites utilizing secondary raw materials for both the matrix (post-industrial rPP) and reinforcing phases (post-consumer cellulose), supplemented with a 3 wt.% commercial MAPP coupling agent to ensure robust interfacial adhesion.

While this study demonstrates the technical viability and lab-scale processability of these circular composite formulations, full commercial drop-in implementation requires further systematic investigation. Future research must evaluate fibre length retention during compounding and moulding, thermo-mechanical degradation across repeated recycling cycles, batch-to-batch property reproducibility, and long-term durability under full-scale industrial trials.

## Figures and Tables

**Table 1:** Physical, morphological, and historical characteristics of the virgin and secondary reinforcing fibres.

Fibre	Origin & Recycling History	Surface Treatment	Moisture Content (%)	Bulk Density (g/cm^3^)	Median Length (µm)	Median Thickness (µm)	Aspect Ratio (L/D)
B600	Virgin hardwood cellulose	None (as-received)	8.26 ± 0.23	0.14 ± 0.01	60	20	3:1
BC300	Virgin hardwood cellulose	None (as-received)	7.09 ± 0.24	0.15 ± 0.01	500	20	25:1
PWC500	Pre-consumer recycled cellulose	None (as-received)	5.79 ± 0.01	0.12 ± 0.01	500	35	14:1
ZZC500	Post-consumer recycled cellulose	None (as-received)	6.57 ± 0.01	0.13 ± 0.01	400	45	9:1
SIS250	Recycled sisal fibre	None (as-received)	8.32 ± 0.06	0.08 + 0.01	1500	300	5:1

**Table 2:** Formulation (wt.%) and coding of composites.

Dosage			Coding of Composites				
rPP	MAPP	Reinforcement	B600	BC300	SIS250	PWC500	ZZC500
100%							
87%	3%	10%	B600-10	BC300-10	SIS250-10	PWC500-10	ZZC500-10
77%	3%	20%	B600-20	BC300-20	SIS250-20	PWC500-20	ZZC500-20
67%	3%	30%	B600-30	BC300-30	SIS250-30	PWC500-30	ZZC500-30

**Table 3:** Thermal degradation temperatures and mass change in composite materials.

Composite	Thermal Degradation		Mass Change			
	T_onD_ (°C)	Δ T_onD_ (%)	T_amb_–225 °C	225–400 °C	400–1000 °C	FR
rPP	274	-	0.3%	67.2%	11.0%	21.5%
B600-10	267	−2.6	0.3%	68.5%	10.8%	20.4%
B600-20	265	−3.3	1.7%	70.0%	11.7%	16.9%
B600-30	262	−4.4	0.3%	71.9%	13.1%	14.7%
BC300-10	265	−3.3	0.5%	68.9%	11.3%	19.3%
BC300-20	265	−3.3	0.9%	70.9%	12.4%	15.7%
BC300-30	264	−3.7	1.1%	71.6%	12.2%	15.0%
SIS250-10	264	−3.7	0.4%	68.4%	11.2%	20.0%
SIS250-20	265	−3.3	1.2%	71.0%	10.5%	17.2%
SIS250-30	266	−2.9	0.7%	72.6%	10.5%	15.2%
PWC500-10	267	−2.6	0.2%	69.6%	10.4%	19.8%
PWC500-20	266	−2.9	0.9%	68.5%	12.1%	18.5%
PWC500-30	264	−3.7	0.9%	69.9%	11.9%	17.3%
ZZC500-10	267	−2.6	0.5%	69.5%	10.2%	19.7%
ZZC500-20	263	−4.0	0.6%	71.8%	10.3%	17.2%
ZZC500-30	260	−5.1	0.7%	71.6%	8.0%	18.7%

**Table 4:** Melting temperature and crystallinity of the polymer matrix and composites reinforced with different fibres.

Composites	Thermal Melting		Crystallinity		
	T_onM_ (°C)	T_M_ (°C)	ΔH_M_ (J/g)	X_C_ (%)	ΔX_C_ (%)
rPP	153	168	42.6	20.4	-
B600-10	154	167	43.3	23.8	16.7
B600-20	153	167	33.2	20.6	1.1
B600-30	155	168	55.0	39.3	92.6
BC300-10	158	170	49.6	27.3	33.9
BC300-20	157	169	41.6	25.8	26.7
BC300-30	154	168	24.9	17.8	−12.9
SIS250-10	154	168	40.8	22.4	10.1
SIS250-20	154	169	41.1	25.6	25.6
SIS250-30	154	168	34.8	24.9	21.9
PWC500-10	154	169	47.3	26.0	27.6
PWC500-20	158	168	38.1	23.6	16.0
PWC500-30	155	168	40.1	28.7	40.6
ZZC500-10	153	168	45.5	25.0	22.8
ZZC500-20	153	167	46.6	29.0	42.1
ZZC500-30	153	167	43.1	30.8	51.0

**Table 5:** Thermal expansion and thermal properties in composite materials.

Composites	Thermal Expansion			Thermal Conductivity			
	dL/L_0,max_ (%)	α_40–120_ (K^−1^)	Δ α_40–120_ (K^−1^)	λ (W/mK)	Δ λ (%)	ε (Ws^1/2^/m^2^·K)	Δ ε (%)
rPP	2.04	146	0.0	0.398 ± 0.002	-	755.9 ± 1.7	-
B600-10	1.73	123	−15.6	0.449 ± 0.004	12.8	806.5 ± 4.3	6.7
B600-20	4.48	128	−12.3	0.488 ± 0.001	22.6	845.6 ± 1.2	11.9
B600-30	4.21	124	−15.2	0.528 ± 0.003	32.7	884.4 ± 2.8	17.0
BC300-10	2.00	131	−10.1	0.474 ± 0.001	19.1	831.0 ± 1.3	9.9
BC300-20	1.99	127	−13.3	0.493 ± 0.003	23.9	849.4 ± 2.5	12.4
BC300-30	1.61	113	−22.7	0.520 + 0.002	30.7	876.9 ± 2.4	16.0
SIS250-10	2.17	136	−6.9	0.465 ± 0.002	16.8	822.1 ± 1.6	8.8
SIS250-20	2.15	137	−5.9	0.482 ± 0.002	21.1	839.5 ± 1.9	11.1
SIS250-30	1.62	109	−25.5	0.506 ± 0.005	27.1	862.9 ± 4.8	14.2
PWC500-10	1.93	141	−3.3	0.430 ± 0.002	8.0	787.7 + 2.4	4.2
PWC500-20	1.81	133	−9.0	0.459 ± 0.003	15.3	816.3 + 3.0	8.0
PWC500-30	1.77	133	−9.0	0.480 ± 0.004	20.6	837.3 ± 4.3	10.8
ZZC500-10	2.19	144	−1.5	0.382 ± 0.002	−4.0	739.6 ± 2.2	−2.2
ZZC500-20	1.93	137	−6.3	0.472 ± 0.003	18.6	829.5 ± 2.6	9.7
ZZC500-30	1.22	104	−28.9	0.514 ± 0.001	29.1	870.4 ± 1.4	15.1

Note: Thermal conductivity (λ) and thermal effusivity (**ε**) were determined from n = 5 transient pulse measurements per sample using the MTPS sensor. The relative standard deviation (RSD %) was below 1.0% across all composite formulations, demonstrating high measurement repeatability.

**Table 6:** Comparison of tensile parameters (Young’s Modulus (Et), Tensile Strength (σ_M_), Elongation at Break (ɛ_t)_) and unnotched Charpy Impact Strength) for composite formulations and reference rPP matrix. Values are expressed as Mean ± SD.

Composite	E_t_ (MPa)	σ_M_ (MPa)	ɛ_t_ (%)	Impact Strength (kJ/m^2^)
rPP (Reference)	2414 ±60	21.8 ± 0.8	3.7 ± 0.9	10.6 ± 0.5
SIS250_10	2691 ± 34	30.6 ± 0.6	2.9 ± 0.4	11.3 ± 0.8
SIS250_20	3140 ± 42	38.2 ± 0.6	3.1 ± 0.1	14.1 ± 0.6
SIS250_30	3492 ± 58	41.8 ± 0.8	2.7 ± 0.3	12.9 ± 1.0
BC300_10	2595 ± 27	27.5 ± 0.5	2.5 ± 0.4	12.4 ± 1.7
BC300_20	2910 ± 35	29.4 ± 0.3	2.4 ± 0.2	11.8 ± 1.5
BC300_30	3363 ± 65	31.2 ± 0.4	2.0 ± 0.2	11.1 ± 1.1
ZZC500_10	2618 ± 39	26.5 ± 1.2	2.3 ± 0.5	9.8 ± 2.7
ZZC500_20	2886 ± 49	28.2 ± 0.6	2.2 ± 0.4	12.9 ± 1.1
ZZC500_30	3166 ± 112	28.8 ± 1.6	2.2 ± 0.5	10.5 ± 1.8
B600_10	2680 ± 26	28.7 ± 0.4	2.6 ± 0.3	15.6 ± 1.1
B600_20	2670 ± 38	24.6 ± 0.2	2.0 ± 0.1	9.0 ± 2.2
B600_30	3012 ± 61	27.5 ± 0.3	1.8 ± 0.1	8.9 ± 0.7
PWC500_10	2547 ± 25	26.3 ± 0.6	2.1 ± 0.2	13.3 ± 2.2
PWC500_20	2776 ± 38	28.5 ± 0.1	2.8 ± 0.4	12.6 ± 0.6
PWC500_30	3075 ± 65	28.4 ± 0.2	2.4 ± 0.3	12.2 ± 0.9

## Data Availability

The original contributions presented in this study are included in the article. Further inquiries can be directed to the corresponding author.

## References

[1] Rumetshofer T., Mager M., Czaker S., Zeilinger H., Fischer J. (2026). Development of a multi-criteria decision tool for the plastics industry—A case study on post-consumer polypropylene packaging material. Sustain. Environ..

[2] Danutė V., Kantautas A., Nizevičienė D., Vaičiukynas Y., Alaburdaitė R. (2024). Utilisation of polypropylene waste and zeolitic by-product in composite materials. Mater. Lett..

[3] European Commission (2020). Action Plan for the Circular Economy: Towards a Cleaner and More Competitive Europe.

[4] Plastics Europe (2023). Plastics—The Facts 2023: An Analysis of European Plastics Production, Demand and Waste Data.

[5] Bressanelli G., Saccani N., Perona M., Baccanelli I. (2020). Towards Circular Economy in the Household Appliance Industry: An Overview of Cases. Resources.

[6] Dey P., Ray S. (2018). An Overview of Recent Trends in the Manufacturing of Green Composites–Considerations and Challenges. Mater. Today Proc..

[7] Bhuiyan M.A.R., Ali A., Akter H., Dayan M., Hossen J., Abden J., Khan A. (2023). Flame resistance and heat barrier performance of sustainable plain-woven jute composite panels for thermal insulation in buildings. Appl. Energy.

[8] Dev B., Rahman M.A., Repon M.R., Rahman M.M., Haji A., Nawab Y. (2023). Recent progress in thermal and acoustic properties of natural fibre-reinforced polymer composites: Preparation, characterisation, and data analysis. Polym. Compos..

[9] Bhardwaj R., Mohanty A.K., Drzal L.T., Pourboghrat F., Misra M. (2006). Renewable resource-based green composites from recycled cellulose fibre and poly(3-hydroxybutyrate-co-3-hydroxyvalerate) bioplastic. Biomacromolecules.

[10] Hansen B., Borsoi C., Dahlem M.A., Catto A.L. (2019). Thermal and thermo-mechanical properties of polypropylene composites using yerba mate residues as a reinforcing filler. Ind. Crops Prod..

[11] Karaoui M., Fiore V., Elhamri Z., Kharchouf S., Alami M., Assouag M. (2025). Study of the physico-chemical properties of injection-Molded polypropylene reinforced with spent coffee grounds. J. Compos. Sci..

[12] Sotohou F., Mwangi J.W., Mutua J.M., Ronoh E.K. (2023). Development and evaluation of recycled polypropylene and bean pod powder composite biomaterial for fused filament fabrication. Adv. Mater. Phys. Chem..

[13] Aberkane A., Bendaikha H., Benzidane R., Jouenne J.B., Alshaikh I.M., Belaadi A., Ghernaout D. (2025). Mechanical, thermal, and morphological properties of recycled PP and HDPE green composites reinforced with atriplex halimus Fibers. J. Nat. Fibers.

[14] Oladele I.O., Falana O.S., Okoro C.J., Onuh L.N., Akinbamiyorin I., Akinrinade S.O., Odemona E.T. (2025). Sustainable composites reinforced with glass fiber and bio-derived calcium carbonate in recycled polypropylene. Hybrid Adv..

[15] Rajak D.K., Pagar D.D., Menezes P.L., Linul E. (2019). Fibre-Reinforced Polymer Composites: Manufacturing, Properties, and Applications. Polymers.

[16] Poletto M. (2019). Natural oils as coupling agents in recycled polypropylene wood flour composites: Mechanical, thermal and morphological properties. Polym. Polym. Compos..

[17] Xie Y., Callum A.S., Xiao Z., Militz H., Mai C. (2010). Silane coupling agents used for natural fibre/polymer composites: A review. Compos. Part A Appl. Sci. Manuf..

[18] Bute I., Tarasovs S., Vidinejevs S., Vevere L., Sevcenko J., Aniskevich A. (2023). Thermal properties of 3D-printed products made from the most common polymers. Adv. Manuf. Technol..

[19] Homkhiew C., Ratanawilai T., Thongruang W. (2015). Composites from recycled polypropylene and rubberwood flour: Effects of composition on mechanical properties. J. Thermoplast. Compos. Mater..

[20] Kada D., Koubaa A., Tabak G., Migneault S., Garnier B., Boudenne A. (2018). Tensile properties, thermal conductivity, and thermal stability of short carbon fibre-reinforced polypropylene composites. Polym. Compos..

[21] Saffian H., Talib M.A., Lee S.H., Md Tahir P., Lee C.H., Ariffin H., Asa’ari A.Z.M. (2020). Mechanical Strength, Thermal Conductivity and Electrical Breakdown of Kenaf Core Fibre/Lignin/Polypropylene Biocomposite. Polymers.

[22] Gall M., Freudenthaler P.J., Fischer J., Lang R.W. (2021). Characterization of composition and structure–property relationships of commercial post-consumer polyethylene and polypropylene recyclates. Polymers.

[23] Bogataj V.Ž., Martínez-García C., Cotes-Palomino M.T., López A.B., Mezek B., Kotnik S. (2026). Recycled sisal fibre-reinforced polypropylene derived from industrial waste stream. Detritus.

[24] (2024). Solid Biofuels—Determination of Moisture Content—Part 2: Simplified Method.

[25] Bogataj V.Ž., Fajs P., Peñalva C., Omahen M., Čop M., Henttonen A. (2019). Utilisation of recycled polypropylene, cellulose and newsprint fibres for the production of green composites. Detritus.

[26] Majeed K., Ahmed A., Abu Bakar M.S., Indra Mahlia T.M., Saba N., Hassan A., Jawaid M., Hussain M., Iqbal J., Ali Z. (2019). Mechanical and Thermal Properties of Montmorillonite-Reinforced Polypropylene/Rice Husk Hybrid Nanocomposites. Polymers.

[27] Chun K.S., Husseinsyah S., Osman H. (2015). Utilization of cocoa pod husk as filler in polypropylene biocomposites: Effect of maleated polypropylene. J. Thermoplast. Compos. Mater..

[28] (2019). Plastics. Determination of Tensile Properties. Part 1: General Principles.

[29] (2012). Plastics. Determination of Tensile Properties. Part 2: Test Conditions for Moulding and Extrusion Plastics.

[30] (2023). Plastics. Determination of Charpy Impact Properties. Part 1: Non-Instrumented Impact Test.

[31] (1975). Testing of Plastics; Impact Test.

[32] Caicedo C., Vázquez Arce A., Crespo L.M., de la Cruz H., Ossa Ó.H. (2015). Material compuesto de matriz polipropileno (PP) y fibra de cedro: Influencia del compatibilizante PP-g-MA. Inf. Téc..

[33] Blanco-Sánchez E., Madera-Mujica A., Pérez-Castillo M., Fajardo-Seminario J., Carrasquero-Rodríguez E., López-López L., Cruz-Riaño L. (2022). Influence of fibre content and annealing on the thermal properties of a bamboo fibre-reinforced biocomposite. Rev. UIS Ing..

[34] Zuhudi N.Z.M., Jayaraman K., Lin R.J.T. (2016). Mechanical, thermal and instrumented impact properties of bamboo fabric-reinforced polypropylene composites. Polym. Polym. Compos..

[35] Khina A.G., Bulkatov D.P., Storozhuk I.P., Sokolov A.P. (2025). Coefficient of linear thermal expansion of polymers and polymer composites: A comprehensive review. Polymers.

[36] Yadav R., Singh M., Shekhawat D., Lee S., Park S.-J. (2023). The role of fillers in enhancing the mechanical, thermal and wear characteristics of polymer composite materials: A review. Compos. Part A Appl. Sci. Manuf..

[37] He Z., Huang S., Lv X., Li J., Zhang G., Sun R. (2023). Novel polyimides with improved adhesion to smooth copper and low coefficient of thermal expansion. Polymer.

[38] Avila-Morales B.B., Ruíz-Jiménez A., Pérez-Ortega F., Caballero-Caballero M., Gutiérrez M. (2026). From agricultural waste to functional composites: Agave bagasse and poly -polypropylene materials with enhanced dimensional and mechanical stability. Fibers Polym..

[39] Singh S.B., Gopalarathnam M., Kodur V.K.R., Matsagar V.A. (2023). Fibre-Reinforced Polymeric Materials and Sustainable Structures.

[40] Rajendran R.C., Rangappa S.M., Puttegowda M., Parameswaranpillai J., Siengchin S., Gorbatyuk S. (2022). Advances in Bio-Based Fibre.

[41] Chávez-Gutiérrez M., De Paoli M.A., Felisberti M. (2012). Biocomposites based on cellulose acetate and short curauá fibres: Effect of plasticisers and chemical treatments of the fibres. Compos. Part A Appl. Sci. Manuf..

[42] Chen Y., Ye L., Dong H. (2023). Lightweight 3D carbon fibre reinforced composite lattice structures of high thermal-dimensional stability. Compos. Struct..

